# Enhanced silicon availability leads to increased methane production, nutrient and toxicant mobility in peatlands

**DOI:** 10.1038/s41598-017-09130-3

**Published:** 2017-08-18

**Authors:** Gloria-Maria Susanne Reithmaier, Klaus-Holger Knorr, Sebastian Arnhold, Britta Planer-Friedrich, Jörg Schaller

**Affiliations:** 1 Environmental Geochemistry, Bayreuth Center for Ecology and Environmental Research (BayCEER), University of Bayreuth, Universitätsstraße 30, 95447 Bayreuth, Germany; 2 Ecohydrology and Biogeochemistry Group, Institute of Landscape Ecology, University of Münster, Heisenbergstr. 2, 48149 Münster, Germany; 3 Ecological Services, Department of Earth Sciences, Bayreuth Center for Ecology and Environmental Research (BayCEER), University of Bayreuth, Universitätsstraße 30, 95440 Bayreuth, Germany

## Abstract

Peatlands perform important ecosystem functions, such as carbon storage and nutrient retention, which are affected, among other factors, by vegetation and peat decomposition. The availability of silicon (Si) in peatlands differs strongly, ranging from <1 to >25 mg L^−1^. Since decomposition of organic material was recently shown to be accelerated by Si, the aim of this study was to examine how Si influences decomposition of carbon and nutrient and toxicant mobilization in peatlands. We selected a fen site in Northern Bavaria with naturally bioavailable Si pore water concentrations of 5 mg/L and conducted a Si addition experiment. At a fourfold higher Si availability, dissolved organic carbon, carbon dioxide, and methane concentrations increased significantly. Furthermore, dissolved nitrogen, phosphorus, iron, manganese, cobalt, zinc, and arsenic concentrations were significantly higher under high Si availability. This enhanced mobilization may result from Si competing for binding sites but also from stronger reducing conditions, caused by accelerated respiration. The stronger reducing conditions also increased reduction of arsenate to arsenite and thus the mobility of this toxicant. Hence, higher Si availability is suggested to decrease carbon storage and increase nutrient and toxicant mobility in peatland ecosystems.

## Introduction

Peatlands are important ecosystems with regard to carbon storage. Although covering only a small fraction of the land surface (3%), they store one third of the world’s soil carbon stock (~550Pg)^1^, which corresponds to half of the CO_2_ in the atmosphere^2^. Natural peatlands also provide the largest natural source of the potent greenhouse gas (GHG) CH_4_, yet, they are considered to have an attenuating effect on global warming by a net carbon fixation^3^, acting as net carbon sinks. However, land use change, such as deforestation, burning, and drainage has turned many peatlands into carbon sources due to e.g. increased peat decomposition^4^. Besides carbon storage, peatlands perform also other ecosystem functions such as nutrient retention and water quality regulation^5^. Nutrient retention and water quality regulation are closely linked as peatlands retain atmospherically or agriculturally deposited nitrogen (N) and phosphorus (P), improving the water quality of downstream ecosystems^6^. Furthermore, peatlands accumulate sulphur^7^, organic pollutants, toxic metals^8^, such as arsenic (As), but release dissolved organic carbon^9^ (DOC) depending on the peat decomposition and hydrology, thus additionally affecting the water quality.

Carbon turnover and nutrient retention in peatlands is a result of the budget of inputs and outputs controlled e.g. by the rate of peat accumulation versus peat decomposition. Among other factors, the peat decomposition rate is influenced by temperature, which controls the activity of decomposing microbes and extracellular enzymes, and by the water table level, which predominantly alternates redox conditions. Under waterlogged, anaerobic conditions peat decomposition is slow, whereas dry aerobic conditions accelerate peat decomposition and cause increased carbon losses^10^. Under anaerobic conditions, elements bound to redox labile sorbents such as iron phases are released into the solution as the sorbents are reduced^11^. This element mobilization due to lowering of the redox potential has been demonstrated also for toxic elements such as As. The As speciation can thereby be used to elucidate dynamics in biogeochemical conditions, because of the shifts in predominant As species depending on changes in redox conditions^12^. Another controlling factor for peat decomposition is the peat nutrient content, which depends on litter quality, but also on exogenous inputs such as atmospheric deposition and input by waters that are enriched by N and P due to agricultural fertilization. Regarding nutrient content of peat, previous research focused mainly on N and P availability as important factors^13^. However, for grass dominated peat ecosystems, silicon (Si) was recently shown to be important for stability of litter, because increased Si contents increase decomposition^14–16^.

Peatlands and other terrestrial ecosystems represent large reservoirs and filters for Si, controlling the Si transfer to the oceans^17^. Land use change during the last 250 years has decreased Si availability in soils by increasing export and decreasing Si storage due to higher erosion and a decrease in vegetation potentially accumulating Si, and has led to a twofold to threefold decrease of the base flow delivery of Si^18, 19^. Due to differences in peat parent material, land use, and peat layer thickness, the Si availability in peatlands is highly variable. Especially in ombrotrophic peatlands, in which mineral weathering and plant growth are mostly decoupled by the peat layer, Si concentrations can be expected to be low in comparison to other ecosystems like forests^18^. Fen ecosystems connected to local groundwater percolating through mineral matter could be expected to plot in an intermediate range. Most available data for Si, however, is based on bulk concentrations derived from X-ray fluorescence analyses (XRF), which are unable to distinguish between bioavailable Si and refractory forms like quartz^20^. Only few studies have analyzed the concentrations of bioavailable Si or pore water Si of peatlands, observing varying concentrations between <1 mg/L and >25 mg/L^21, 22^. In field and laboratory experiments it was found that plants grown under high Si availability showed a higher decomposition rate^14–16^. This suggests that Si may influence the ecosystem function of peatlands, especially in grass dominated fens with silicon being beneficial for grasses^23^, by accelerating the peat decomposition. Higher peat decomposition rates could increase GHG production and decrease the binding capacity of the peat for nutrients and other elements. However, so far, the effect of Si availability carbon turnover in peatlands is not well known.

Consequently, the aim of this study was to investigate how Si availability influences the biogeochemistry of peatlands. We hypothesize that increased Si availability (i) increases production of CO_2_ and CH_4_ as well as DOC release, (ii) mobilizes nutrients, such as N and P, because of competition for binding sites, and (iii) deteriorates water quality by mobilizing potentially toxic trace elements, such as As, due to changes in redox conditions. To this end, a field experiment was conducted at a minerotrophic fen site in Bavaria, Germany. Pore water samples and dissolved gases were analyzed under low and high Si availabilities to identify the effect of Si on CO_2_ and CH_4_ production and nutrient and trace element mobilization.

## Results

### Influence of Si availability on conductivity, CO_2_, CH_4_, and DOC in pore water

**Figure 1:**
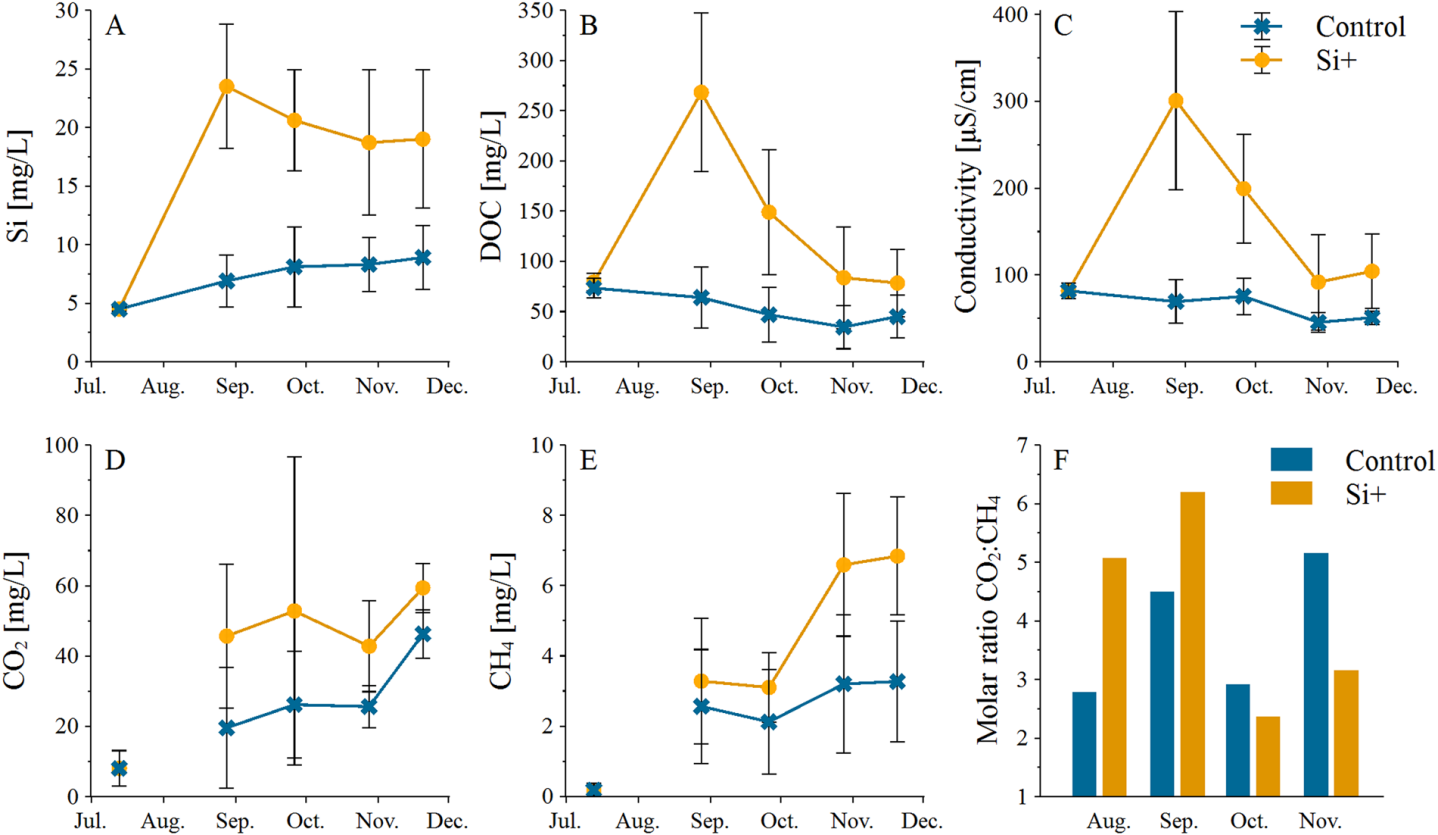
Mean ± SD values of Si, DOC, CH_4_, and CO_2_ concentrations, conductivity, and the molar ratio CO_2_:CH_4_ in pore water under high (Si+) and low (control) Si availability, n = 4.

One month after Si was added, average Si concentrations were threefold higher in the Si+ treatments than in the controls (Fig. 1A). The two treatments (control and Si+ treatment) resulted in significantly different conductivities and pore water concentrations of DOC (*p* ≤ 0.001), CH_4_ (*p* ≤ 0.001), and CO_2_ (*p* = 0.003). With Si addition, DOC concentrations and conductivity exceeded values of the controls by a factor of four one month after Si addition and decreased to about the initial values after three months (Fig. 1B and C). In the controls, DOC and conductivity did not show a temporal pattern, but remained more or less constant. Concentrations of CO_2_ and CH_4_ of the first measurement were close to zero, probably, because equilibrium between pore water and gas phase in the gas samplers was not reached, yet. These first values were excluded from further interpretation (Fig. 1D and E). In the following months, high Si availability increased both, CO_2_ and CH_4_ concentrations_,_ by an average factor of 1.7. However, the difference between high and low Si availability decreased for CO_2_ and increased for CH_4_ over time. Since CO_2_ concentrations were considerably higher than CH_4_ concentrations (up to 6-fold), the molar CO_2_:CH_4_ ratio in both treatments was always larger than one (Fig. 1F). In August and September, the CO_2_:CH_4_ ratio under high Si availability was higher than under low Si availability, whereas in October and November it was lower. In addition, we found minor differences (ΔpH < 1) in pore water pH between both treatments at the end of the experiment. These differences, however, did not exceed previously reported natural variability due to redox dynamics at the study site^24^, which had only minor effects on element mobilization. Furthermore, no significant correlation between differences in pH and any other investigated parameter was found (data not shown). We therefore deduce that pH is no relevant parameter for explaining the observed differences between high and low Si availability.

### Influence of Si availability on the element concentrations in pore water

**Figure 2:**
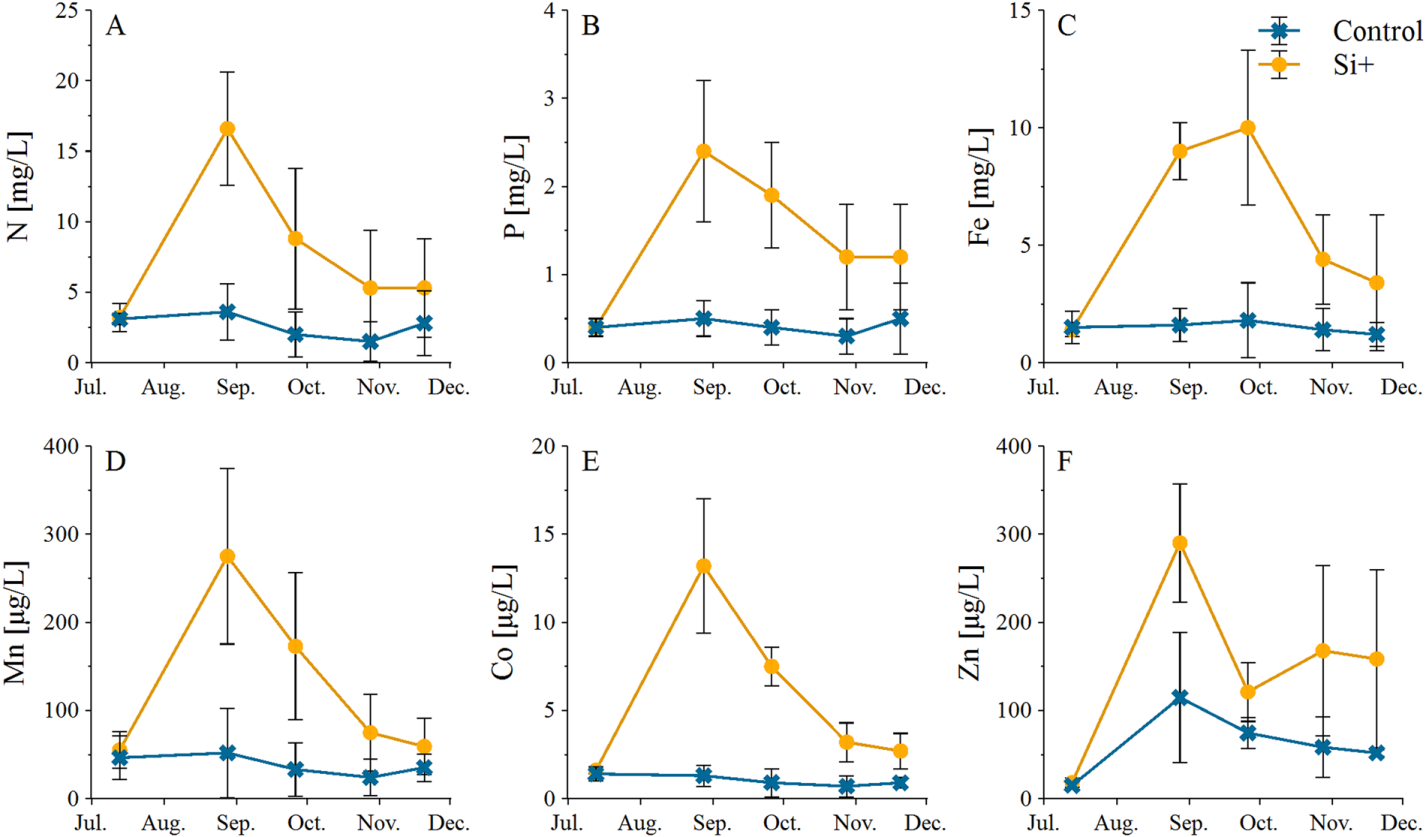
Mean ± SD values of N, P, Fe, Mn, Co, and Zn concentrations in pore water under high (Si+) and low (control) Si availability, n = 4. Note that N, P, and Fe concentrations are plotted in mg/L, whereas Mn, Co, and Zn are plotted in µg/L.

Concentrations of total dissolved N, P, Fe, Mn, Co, and Zn in pore water were significantly higher under high compared to low Si availability (*p* ≤ 0.001 each, Fig. 2). Apart from Fe, which peaked two months after Si addition, all elements had highest concentrations immediately after Si addition. The highest increase after Si addition was observed for Co (10-fold), followed by Fe (6-fold), and both N and P (both 5-fold). Note that after the elements had reached a temporary maximum, concentrations decreased faster towards initial values than Si concentrations did. Only Zn did not show a continuous decrease, but remained rather stable at elevated concentrations. Under low Si availability, all element concentrations, except for Zn (p < 0.05, ANOVA), did not significantly differ during the experiment (n.s., ANOVA). Remarkably, Si and P concentrations of individual measurements in pore water were highly correlated (Pearson r = 0.89) (Figs 1, 2).

**Figure 3:**
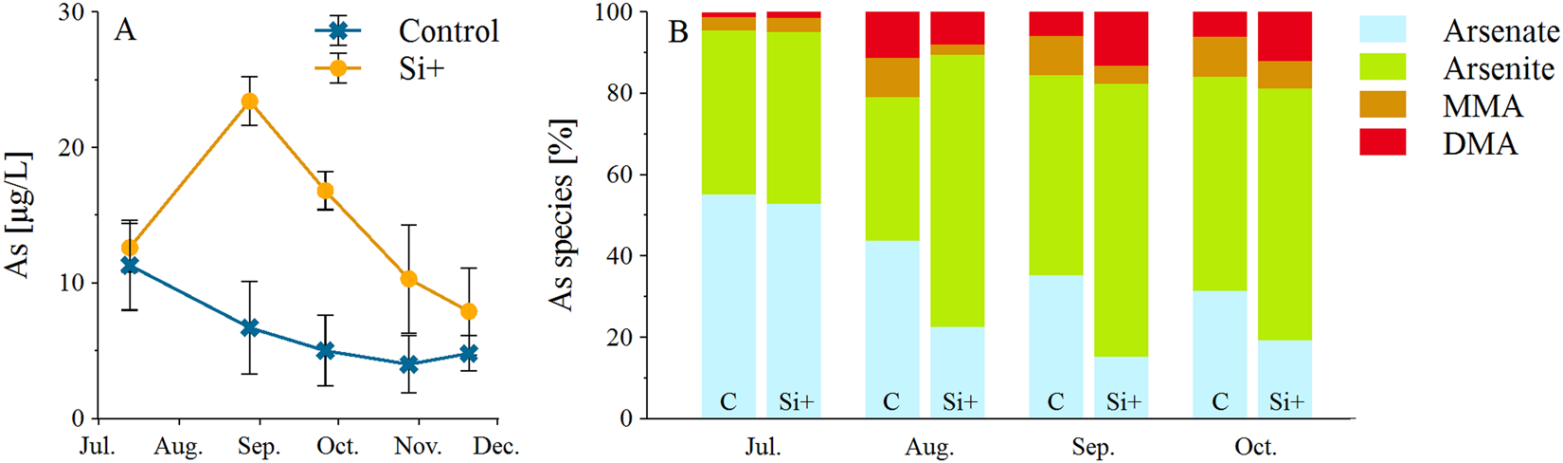
Mean ± SD values of total As and percentage share of As species in pore water under high (Si+) and low (control) Si availability, n = 4.

Total As concentrations were also elevated under high Si availability (Fig. 3A). Due to problems with sample preservation, As speciation could not be measured for the last sampling, i.e., four months after Si addition. However, the last available sample suggested a converging trend in concentrations of the As species of both treatments were, differences were statistically insignificant (n.s., *t*-test) (Fig. 3B). At the beginning of the experiment, directly after the installation of the pore water samplers, arsenate concentrations were higher and organic As species were less abundant than in the following months. In both treatments, inorganic As species dominated (79–95%). Under high Si availability ~70% more arsenite and ~48% less arsenate occurred compared to low Si availability. Furthermore, one month after the Si addition less organic As species occurred in the Si+ treatments, which was reversed thereafter. Under low Si availability, MMA was the dominant organic As species, whereas under high Si availability, DMA was more abundant. Interestingly, in some cases under high Si availability minor amounts of mono-, di-, and trithioarsenate occurred (<0.8 µg/L). Details about the As species (Table S1), general soil characteristics (Table S2), weather data (Figure S1), pH, and temperature in pore water (Figure S2) can be found in the supporting information.

## Discussion

### Silicon controls on carbon turnover

The increased concentrations of CO_2_, CH_4_, and DOC in pore water under high Si availability clearly confirm the relevance of Si for the carbon cycle and support hypothesis (i) that increased Si availability increases concentrations of CO_2_ and CH_4_ in the peat and leads to a release of DOC into porewater (Fig. 1). Assuming otherwise similar conditions of diffusivity and transport as in the control, higher concentrations of CO_2_ and CH_4_ would indicate higher emissions of these gases and thus higher rates of decomposition. Moreover, a higher release of DOC into the porewater constitutes another potentially mobile fraction of carbon and thus increased losses of carbon from the system due to increased Si availability. We are aware that increased concentrations would not necessarily be a proof for increased production rates. However, assuming that diffusivity, peat properties, and water saturation of treatment and control remained similar during out experiment and assuming a steady state of production and diffusive emission, increased concentration levels can be interpreted as a result of higher production of CO_2_ and CH_4_ in the peat. Since those three components (CO_2_, CH_4_ and DOC) represent products of organic matter decomposition^10^ Si seems to accelerate decomposition and therefore presumably influences the carbon turnover of such ecosystems. This is in line with the findings of Schaller and Struyf^14^, who showed a positive correlation of plant litter Si concentration and microbial decomposition rate in a laboratory experiment. The same positive effect of Si on litter decomposition was also proven in a field experiment^15^. Since in our study plants were not present in the mesocosms, direct plant effects on CO_2_ formation via rhizosphere respiration could be excluded. Consequently, heterotrophic respiration should be the main source of the surplus of CO_2_^25^. The increase in CO_2_, CH_4_ and DOC concentration in porewaters could thus be explained by Si mobilizing elements like P into pore waters, due to competition of Si and P for binding sites as already suggested for As^26^ (Fig. 2). P availability however, interferes with C turnover, as P is known to compete with C for binding sites^27^ and thus affects the C turnover by increasing pore water DOC concentrations, making organic carbon more accessible for decomposition. Since the molar CO_2_:CH_4_ ratio was always higher than one, conditions in the mesocosms were not strictly methanogenic^28^, but high Si availability temporarily decreased CO_2_:CH_4_ ratios (Fig. 1), confirming the effect of Si addition leading to lower redox conditions. Interestingly, high Si availability increased CO_2_ production at first. Thereafter, upon depletion of thermodynamically favorable electron acceptors, like nitrate and ferric iron, methanogenesis increased under high Si availability^28^. Simultaneously, decomposition led to a reductive dissolution of redox labile phases, and mobilized DOC and associated elements^24^. This release increased the ionic strength in Si+ treatments as shown before^11^. In summary, high Si availability increased CO_2_ as well as CH_4_ production and DOC release in fens and favored the rapid onset of reducing conditions.

Enhanced decomposition can not only increase CO_2_ and CH_4_ concentrations in pore water, but can also increase outgassing of these GHGs. The extent of the outgassing depends on production, consumption, and transport of the GHGs in pore water, which were, however, not studied in further detail here. In general, outflow, diffusion, advection, bubble formation, and direct transfer by vascular plants are the possible transport mechanisms of CO_2_ and CH_4_^29–31^. Even though in the present study some of the CH_4_ was probably reoxidized to CO_2_^32^_,_ and the fate of CH_4_ was not studied in detail, three months after Si addition, the CH_4_ concentrations were high enough to form bubbles (>390 µM equivalent to CH_4_ partial pressures around 0.2 atm) followed by outgassing^33^. Hence, an increased GHG production is supposed to increase GHG emissions under elevated Si availability.

### Silicon controls on nutrient mobilization

High Si availability considerably increased concentrations of N and P in pore water (Fig. 2), confirming hypothesis (ii) that Si mobilizes nutrients. This higher release of N and P into the pore water may be due to dissolved Si competing with other elements for binding sites at organic matter and mineral surfaces. This process was already suggested by Seyfferth and Fendorf for As^26^, who determined that Si effectively competes with P for binding sites of Fe and Al(hydr)oxides in rice paddy fields and Neu *et al*. showing a higher P uptake by plants under high Si availability suggesting elevated P mobility^23^. The fact that Si highly correlated with P in our experiment strongly supports that P is displaced by Si at the binding sites. From previous studies it is known that at this particular fen site, but likely also in other fens, iron oxides are present in notable quantities^34^. Additional to the competition at binding sites, Si also apparently accelerated the mineralization of organic matter, as obvious from increases in CO_2_ and CH_4_, causing additional N and P release from decomposition into the pore water^35^. The fact that N and P concentrations decreased towards initial concentrations within a few months after the Si application may have partly resulted from increased microbial assimilation of nutrients^36^. However, leaching might also have been effective due to high runoff rates after intensive precipitation events at the study site^9^ (Figure S1). Most probably some of the Si also leached out, but the dissolution of Si from the Si-nanoparticle addition (amorphous Si), which remained in suspension, may have buffered the Si leaching^37^.

Besides the macronutrients N and P, also the concentrations of micronutrients and potentially toxic elements such as Fe, Mn, Co, Zn, and As increased in pore water after Si addition (Fig. 3), confirming hypothesis (iii) that increased Si availability can deteriorate water quality by mobilizing potentially toxic trace elements. Similar to N and P, these elements may have been replaced by Si at the binding sites, and as a result the re-adsorption may be controlled by the competition between these elements^26^. Additionally, Fe, Mn, Co, and As are redox-sensitive, as already mentioned above. Therefore, their release might be driven by the more rapid and stronger decrease in redox conditions towards methanogenesis under high Si availability. Consequently, elements bound to ferric iron phases and DOC co-precipitated with iron were released into solution^11, 38^. In comparison to the other studied elements, the decrease of Fe was delayed hinting to ongoing reductive dissolution of a large pool of iron phases under continuously reduced conditions^39^. Moreover, the dissolution of Fe and Mn (hydro)oxides may have released bound As and P into solution^40^. Due to the high activity of sulfate reduction and subsequent formation of sulfides at the site the potential of As and P re-adsorption is low and the elements remain in solution^41^. The shift towards reducing conditions was also reflected in the increase of the reduced As species arsenite under high Si availability (Fig. 3). Furthermore, the continuous methylation from MMA to DMA under high Si availability conditions occurred simultaneously to the increased activity of methanogens, which are able to methylate As^42^. Lafferty and Loeppert^43^ showed a decrease of As adsorption at Fe (hydr)oxide surfaces by increased methyl substitution of As. This may explain the increased As release under high Si availability. Nevertheless, high Si availability did not only increase total As concentrations but also released higher concentrations of the more toxic and mobile arsenite into the pore water^44^. These observations also demonstrate that the analysis of As speciation provided additional insight into the particular biogeochemical conditions, i.e., with Si favoring strongly reducing conditions with concomitant sulfate reduction and methanogenesis.

**Figure 4:**
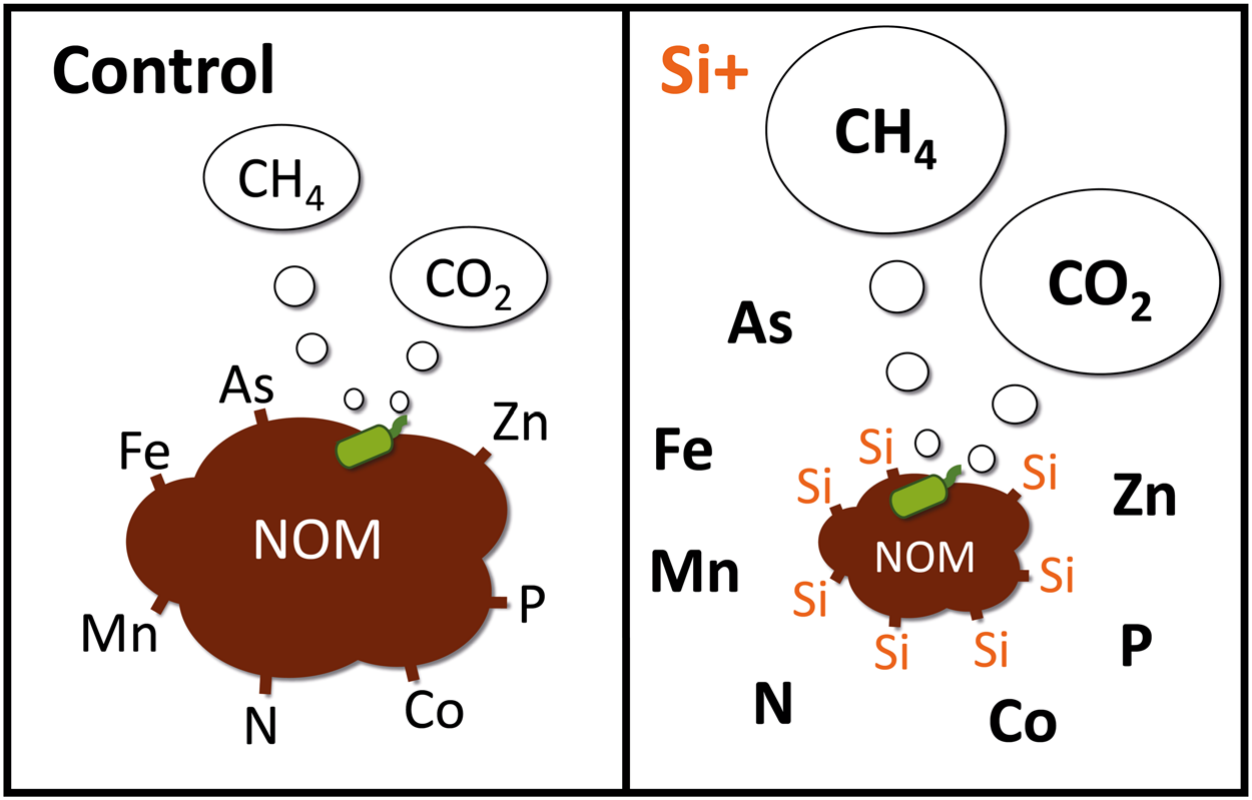
Overview of Si effects on peatland biogeochemistry, element binding to natural organic matter (NOM) and production of CO_2_ and CH_4_.

In conclusion, Si availability among different peatlands is highly different and thus may indeed exert an important control on biogeochemical processes as observed in our study. Our results revealed that high Si availability leads to N and P mobilization (Fig. 4). However, high Si availability in fens is potentially accelerating organic matter decomposition by increasing CO_2_ and especially CH_4_ production (Fig. 4). Competing with other elements, such as N, P, and As, for binding sites and promoting reducing conditions, high Si availability causes an enhanced nutrient and toxicant mobilization into solution (Fig. 4). In summary, Si exerts an important and so far little considered control on carbon and nutrient turnover in peatlands.

## Methods

### Sampling site

The Si addition experiment was conducted at the “Schlöppnerbrunnen fen site”, which is located in the Lehstenbach catchment (4.2 km^2^) in northeastern Bavaria (50°07′56.8″ N, 11°52′55.5″ E, 697 m ASL). The study area is characterized by a continental temperate climate with a mean annual precipitation of 1038 mm (1994–2015) and mean annual temperature of 6.5 °C (1994–2015). The minerotrophic peatland has a central open area, which is surrounded by a spruce forest (Picea abies). The open area is covered by grasses and bryophytes^45^. The thickness of the peat varies from 30 to 120 cm and the organic material is highly decomposed below 5–10 cm depth^46^. The site has been intensively studied previously with regard to carbon turnover and redox processes (e.g. refs 34, 47), carbon export^24^, CO_2_ exchange^48^, and microbiology (e.g. ref. 49). During the experiment, the peat was waterlogged and the water tables were constantly 2 cm above the soil surface.

### Experimental setup

The experiment was set up in July 2015. Eight mesocosms (PVC tubes, length: 40 cm, diameter: 15 cm) were installed in pairs (each containing one control and one treatment with Si addition about 10 cm apart from each other) randomly selected within homogeneous but sparse vegetation of *Carex rostrata*. However, those plants died due to installation of the tubes before the start of the experiment. Soil samples from the top peat layer were taken close to the pairs of mesocosms. Each pair consisted of a mesocosm to which 50 g of low acidic (pH ~ 4.7), synthetic amorphous silica (Aerosil 300; Evonik Industries AG) was added, which is equal to 23 g of elemental Si, suspended in 1 L deionized water (Si+ treatment) and a control watered with 1 L deionized water. The synthetic amorphous silica is highly dissolvable even at lower pH and is an analogue to nano particulate volcanic ash. The Si was added only once at the beginning of the experiment.

### Field sampling

Pore water and gas samples were obtained from each mesocosm on a monthly basis between July and November, with the first sampling starting directly before Si addition to the Si treatment. To this end, gas samplers (polysiloxane tubes allowing diffusive equilibration, length: 20 cm, diameter: 1 cm) were installed in the top peat layer just below the surface. The polysiloxane tubes were connected to impermeable polyurethane tubes and closed by three-way stopcocks for sampling following a sampling procedure described previously^50^. The gas samples were taken with plastic syringes (3 ml, Omnifix, Braun). Pore water was sampled by polyvinylchloride tubes (length: 50 cm, diameter: 1 cm), which were installed in the top peat layer of each mesocosm. The lowest 15 cm of the tubes, which were placed in the top peat layer just below the surface, were perforated at 5 mm intervals. To eliminate coarse particles, the tubes were covered by nylon prefilters. The pore water samples were taken by syringes to measure pH, temperature (HQ40d Multi, PHC101 electrode, Hach), and conductivity (Winlab, Windaus). For the analysis of total element and species concentrations, all water samples were filtered after extraction (0.2 µm, cellulose-acetate filter, Chromafil). To prevent precipitation of sulfide or iron minerals, samples for total element analysis 150 µL of H_2_O_2_ (analytical grade, 30%, Fisher Scientific) and 250 µL HNO_3_ (analytical grade, 70%, Fisher Scientific) were added per 10 mL sample. Samples used for total N and DOC measurement were frozen after the sampling. Samples for species analysis of As were preserved by immediate flash-freezing in dry ice on site.

### Laboratory analysis

Concentrations of CO_2_ and CH_4_ in the gas samples, which were obtained from the diffusive equilibration samplers, were measured within three hours after the sampling by a gas chromatograph (SRI 8610 C equipped with methanizer and flame ionization detector, SRI Instruments). Concentrations of the dissolved gas in the pore water were recalculated applying the Henry’s law and a temperature correction for *in-situ* temperatures^51^. The soil samples were freeze-dried and then ground to fine powder with a zirconium ball mill. Subsequently soil samples were digested in 3 ml of HNO_3_ and 2 ml H_2_O_2_ using a CEM Mars5 microwave digestion system (CEM Corporation, Matthews, NC, USA). An ICP-OES (Vista – Pro, Varian Inc./ Cetac) was used to measure total Si concentrations in the pore water samples and in the soil samples after extraction in a 0.1 M Na_2_CO_3_ solution at 85 °C for five hours according to Struyf, *et al*.^52^. Total dissolved element concentrations in pore water and soil extracts of P, manganese (Mn), iron (Fe), cobalt (Co), zinc (Zn), and As were analyzed by a quadrupole ICP-MS (X-Series2, Thermo Scientific). The elements Mn, Fe, Co, and Zn were measured in KED mode (kinetic energy discrimination, 3 V energy discrimination for Fe und 2 V for Mn, Co, and Zn, 93% helium and 7% hydrogen as collision gas), whereas P and As were measured in oxygen mode (10% oxygen in helium as a reaction gas). Total element concentrations of N and DOC concentrations in pore water were measured with a TOC-Analyzer (TOC-V CPN, Shimadzu Corporation) with an integrated total N measuring unit. The As speciation analysis was done using an ion chromatography (IC Dionex 3000 with a AG16/AS16 on Ion-Pac column, 4 mm, Thermo Scientific) coupled to a quadrupole ICP-MS (X-Series2, Thermo Scientific). An alkaline eluent with a gradient of 2.5–100 mM NaOH was used at a constant flow rate of 1.2 mL/min as described before^53^. For quantification, commercially available salts of arsenite, arsenate, monomethylarsenate (MMA), and dimethylarsenate (DMA) were used. Mono-, di- and trithioarsenate were quantified via the arsenate calibration. The validity of this approach has been confirmed before^53^.

### Statistical analysis

A *t*-test and a multifactorial analysis of variance (ANOVA) of the factors treatment (high and low Si availability) and time (month) were applied using SPSS version 16.01.

## Electronic supplementary material

Supplementary Information
