# Supplementary material for: Enhanced silicon availability leads to increased methane production, nutrient and toxicant mobility in peatlands
Source: Sci Rep. 2017 Aug 18;7:8728. doi: 10.1038/s41598-017-09130-3 (PMC5562759; doi:10.1038/s41598-017-09130-3)
Supplement: Supplementary file 1 — Supplementary Information [file 41598_2017_9130_MOESM1_ESM.doc]

Supporting Information to

**Enhanced silicon availability leads to increased methane production, nutrient and toxicant mobility in peatlands**

Authors: Gloria-Maria Susanne Reithmaier^1^, Klaus-Holger Knorr^2^, Sebastian Arnhold^3^, Britta Planer-Friedrich^1^, Jörg Schaller^1*^

^1^ *Environmental Geochemistry, Bayreuth Center for Ecology and Environmental Research (BayCEER), University of Bayreuth, Universitätsstraße 30, 95447 Bayreuth, Germany*

^2^ *Ecohydrology & Biogeochemistry Group, Institute of Landscape Ecology, University of Münster, Heisenbergstr 2, 48149 Münster, Germany*

^3^ *Ecological Services, Department of Earth Sciences, Bayreuth Center for Ecology and Environmental Research (BayCEER), University of Bayreuth, Universitätsstraße 30, 95440 Bayreuth, Germany*

- 3 Pages
- 2 Tables
- 2 Figures

*^*^*corresponding author: phone +49 0921 553991, fax +49 0921 552334;

email: joerg.schaller@uni-bayreuth.de

**Table S1:** Components of arsenic speciation in pore water in Si+ treatments and controls (C), n = 4. The concentrations of the dead volume (DV), dimethylarsenate (DMA), an unknown peak after 370 s (UN1), monomethylarsenate (MMA), arsenate, mono-, di- and trithioarsenate (Mono, Di, Tri) were measured by ICP-MS. Concentrations of MMA and DMA were determined by individual calibration curves. Concentrations of DV and the unknown peaks were calculated using the arsenite calibration as the peaks were closest to arsenite, whereas methylated and thiolated arsenates were analyzed by using arsenate calibration.

|  | **N°** | **Tr.** | **DV [µg/L]** | **DMA [µg/L]** | **UN1 [µg/L]** | **Arsenite [µg/L]** | **UN2 [µg/L]** | **MMA [µg/L]** | **Arsenate [µg/L]** | **Mono [µg/L]** | **Di [µg/L]** | **Tri [µg/L]** |
| --- | --- | --- | --- | --- | --- | --- | --- | --- | --- | --- | --- | --- |
| 7/13/2015 | 1 | C | 0.12 | 0.08 | 0.02 | 2.53 | 0.04 | 0.33 | 5.36 | 0.00 | 0.00 | 0.00 |
|  |  | Si+ | 0.07 | 0.05 | 0.07 | 2.06 | 0.06 | 0.28 | 3.53 | 0.07 | 0.13 | 0.00 |
|  | 2 | C | 0.12 | 0.07 | 0.06 | 1.08 | 0.00 | 0.32 | 2.86 | 0.00 | 0.00 | 0.00 |
|  |  | Si+ | 0.21 | 0.10 | 0.00 | 2.06 | 0.00 | 0.22 | 3.04 | 0.00 | 0.00 | 0.00 |
|  | 3 | C | 0.21 | 0.12 | 0.00 | 4.77 | 0.00 | 0.29 | 3.63 | 0.00 | 0.00 | 0.00 |
|  |  | Si+ | 0.24 | 0.24 | 0.00 | 3.73 | 0.00 | 0.35 | 5.66 | 0.00 | 0.00 | 0.00 |
|  | 4 | C | 0.26 | 0.14 | 0.00 | 5.09 | 0.00 | 0.22 | 6.71 | 0.00 | 0.00 | 0.00 |
|  |  | Si+ | 0.22 | 0.13 | 0.00 | 5.55 | 0.00 | 0.22 | 4.57 | 0.00 | 0.00 | 0.00 |
| 8/28/2015 | 1 | C | 0.25 | 0.56 | 0.08 | 1.04 | 0.00 | 0.26 | 1.59 | 0.00 | 0.00 | 0.00 |
|  |  | Si+ | 1.53 | 0.64 | 0.31 | 5.12 | 0.05 | 0.23 | 4.61 | 0.76 | 0.55 | 0.21 |
|  | 2 | C | 0.81 | 0.50 | 0.19 | 0.63 | 0.00 | 0.23 | 1.04 | 0.00 | 0.00 | 0.00 |
|  |  | Si+ | 2.12 | 0.98 | 0.00 | 6.24 | 1.08 | 0.29 | 1.64 | 0.00 | 0.05 | 0.00 |
|  | 3 | C | 0.18 | 0.08 | 0.33 | 0.58 | 0.22 | 0.23 | 0.78 | 0.00 | 0.00 | 0.00 |
|  |  | Si+ | 1.44 | 1.09 | 0.00 | 8.63 | 0.95 | 0.23 | 1.40 | 0.00 | 0.00 | 0.00 |
|  | 4 | C | 0.07 | 0.00 | 0.20 | 0.51 | 0.19 | 0.23 | 1.00 | 0.00 | 0.00 | 0.00 |
|  |  | Si+ | 1.59 | 0.50 | 0.00 | 6.30 | 0.39 | 0.27 | 1.33 | 0.00 | 0.09 | 0.00 |
| 9/26/2015 | 1 | C | 0.13 | 0.18 | 0.24 | 1.06 | 0.13 | 0.24 | 0.84 | 0.00 | 0.00 | 0.00 |
|  |  | Si+ | 1.45 | 0.93 | 0.00 | 4.40 | 0.06 | 0.27 | 0.82 | 0.00 | 0.00 | 0.00 |
|  | 2 | C | 0.63 | 0.25 | 0.74 | 1.19 | 0.00 | 0.24 | 1.01 | 0.00 | 0.00 | 0.00 |
|  |  | Si+ | 0.98 | 0.85 | 1.69 | 0.65 | 0.18 | 0.24 | 0.95 | 0.00 | 0.00 | 0.00 |
|  | 3 | C | 0.13 | 0.06 | 0.22 | 0.71 | 0.08 | 0.24 | 0.88 | 0.00 | 0.00 | 0.00 |
|  |  | Si+ | 0.51 | 0.52 | 3.18 | 0.70 | 0.72 | 0.24 | 0.81 | 0.00 | 0.00 | 0.00 |
|  | 4 | C | 0.10 | 0.10 | 0.14 | 0.61 | 0.00 | 0.24 | 0.79 | 0.00 | 0.00 | 0.00 |
|  |  | Si+ | 1.25 | 0.63 | 0.00 | 4.18 | 0.03 | 0.24 | 0.74 | 0.00 | 0.00 | 0.00 |
| 10/28/2015 | 1 | C | 0.18 | 0.19 | 0.17 | 1.05 | 0.00 | 0.24 | 0.99 | 0.00 | 0.00 | 0.00 |
|  |  | Si+ | 1.39 | 0.92 | 1.84 | 1.56 | 0.10 | 0.24 | 0.77 | 0.00 | 0.00 | 0.00 |
|  | 2 | C | 0.49 | 0.35 | 0.99 | 0.96 | 0.09 | 0.24 | 0.70 | 0.00 | 0.00 | 0.00 |
|  |  | Si+ | 0.44 | 0.22 | 0.85 | 0.83 | 0.14 | 0.24 | 0.65 | 0.00 | 0.00 | 0.00 |
|  | 3 | C | 0.11 | 0.05 | 0.21 | 1.02 | 0.00 | 0.24 | 0.64 | 0.00 | 0.00 | 0.00 |
|  |  | Si+ | 0.07 | 0.10 | 0.20 | 0.67 | 0.15 | 0.24 | 0.60 | 0.00 | 0.00 | 0.00 |
|  | 4 | C | 0.00 | 0.00 | 0.06 | 0.64 | 0.00 | 0.24 | 0.70 | 0.00 | 0.00 | 0.00 |
|  |  | Si+ | 0.99 | 0.49 | 1.23 | 1.54 | 0.00 | 0.24 | 0.69 | 0.00 | 0.00 | 0.00 |

**Table S2: Average element concentrations of soil samples and standard deviation (Std), n=4.**

| **Element** | **Si** | **P** | **Mn** | **Fe** | **Co** | **Zn** | **As** |
| --- | --- | --- | --- | --- | --- | --- | --- |
| Average±Std [mg/kg] | 27000±4900 | 6900±937 | 43±35 | 3000±1200 | 3±3 | 62±46 | 19±10 |


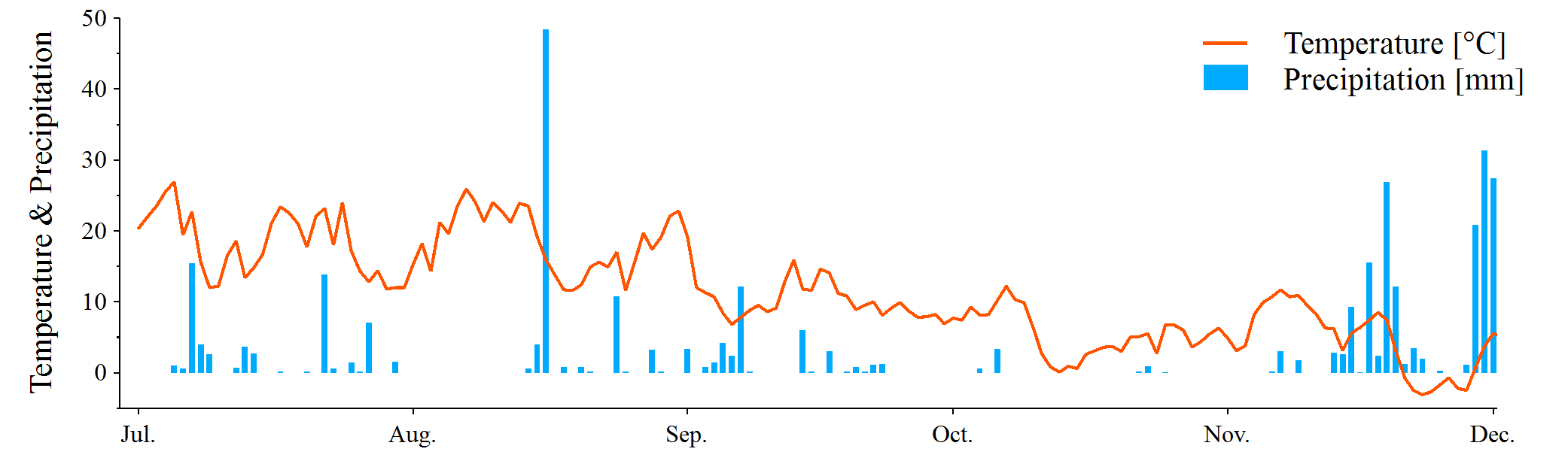


**Figure S1:** Average daily temperature and sum of the daily precipitation at the Schlöppnerbrunnen fen site during the experimental period in 2015.


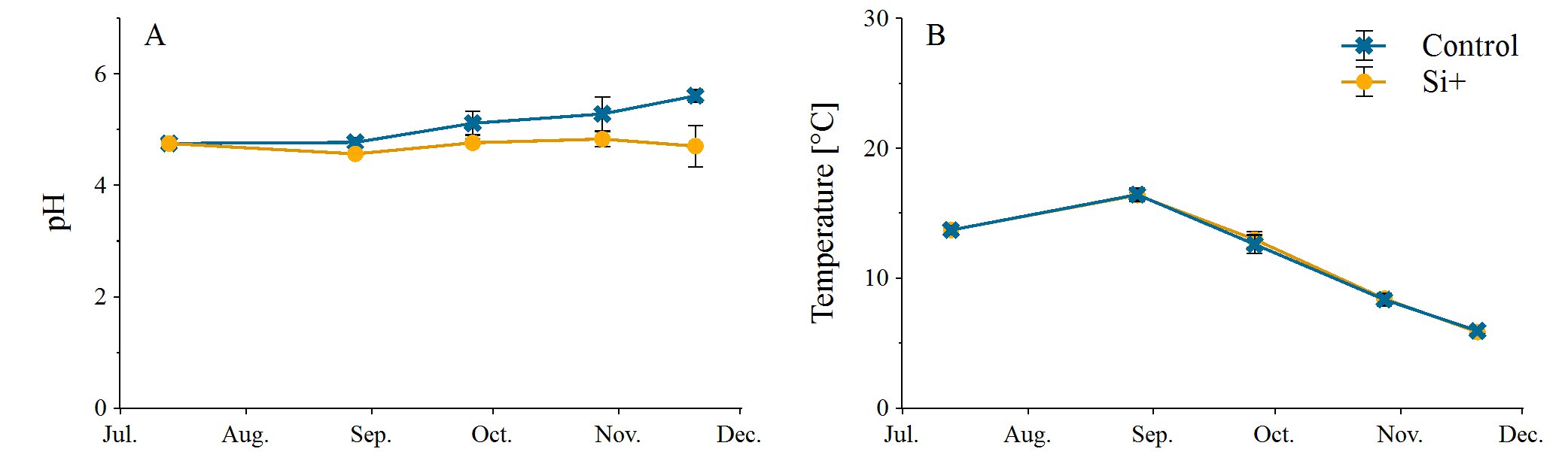
**Figure S2:** Mean±SD values of pH and temperature in pore water under high (Si+) and low (control) Si availability, n=4.
